# The design and statistical power of treatment re-infection studies of the association between pre-erythrocytic immunity and infection with *Plasmodium falciparum*

**DOI:** 10.1186/1475-2875-12-278

**Published:** 2013-08-08

**Authors:** Michael T White, Jamie T Griffin, Azra C Ghani

**Affiliations:** 1 MRC Centre for Outbreak Analysis and Modelling, Department of Infectious Disease Epidemiology, Imperial College London, Norfolk Place, London W2 1PG, UK

## Abstract

**Background:**

Understanding the role of pre-erythrocytic immune responses to *Plasmodium falciparum* parasites is crucial for understanding the epidemiology of malaria. However, published studies have reported inconsistent results on the association between markers of pre-erythrocytic immunity and protection from malaria.

**Methods:**

The design and statistical methods of studies of pre-erythrocytic immunity were reviewed, and factors affecting the likelihood of detecting statistically significant associations were assessed. Treatment re-infection studies were simulated to estimate the effects of study size, transmission intensity, and sampling frequency on the statistical power to detect an association between markers of pre-erythrocytic immunity and protection from infection.

**Results:**

Nine of nineteen studies reviewed reported statistically significant associations between markers of pre-erythrocytic immunity and protection from infection. Studies with large numbers of participants in high-transmission settings, followed longitudinally with active detection of infection and with immune responses analysed as continuous variables, were most likely to detect statistically significant associations. Simulation of treatment re-infection studies highlights that many studies are underpowered to detect statistically significant associations, providing an explanation for the finding that only some studies report significant associations between pre-erythrocytic immune responses and protection from infection.

**Conclusions:**

The findings of the review and model simulations are consistent with the hypothesis that pre-erythrocytic immune responses prevent *P. falciparum* infections, but that many studies are underpowered to consistently detect this effect.

## Background

There are several vaccines targeting the pre-erythrocytic stages of the *Plasmodium falciparum* parasite under development [1-3] that aim to confer protection by boosting the pre-erythrocytic (PE) immune response to levels much higher than observed under conditions of natural exposure, or inducing sterile immunity in malaria naïve individuals. Recent advances in vaccine development have led to renewed interest in the PE immune response [4], both naturally acquired and vaccine-induced. Despite the considerable progress in the vaccine development effort, the association between naturally acquired PE immune responses and protection from infection remains poorly understood.

When a *P. falciparum* infectious *Anopheles* mosquito bites a human, sporozoites are inoculated into the tissue surrounding the injection site [5], where they migrate to blood vessels [6] from where they are carried to the liver. Upon reaching the liver, sporozoites invade hepatocytes, differentiate into liver-stage parasites and release merozoites into the blood a few days later [7]. The PE immune response can prevent successful sporozoite development via antibody- or cell-mediated responses targeting sporozoite antigens: the prime targets being circumsporozoite protein (CSP), thrombospondin-related adhesion protein (TRAP) and liver-stage antigen 1 (LSA-1) [8]. The mechanisms underlying antibody-mediated protection include inhibition of hepatocyte invasion, opsonization of sporozoites for uptake by macrophages and dendritic cells, and possibly a reduction in the infectious dose of sporozoites [9]. Cell-mediated immunity is provided by CD4^+^ or CD8^+^ T-cells, both of which have been observed to eliminate infected hepatocytes *in vitro*[10,11].

The failure of sporozoite inoculations to consistently progress to blood-stage malaria has been observed in both artificial challenge studies [12] and under natural exposure [13-15]. The proportion of infectious mosquito bites progressing to blood-stage infection has been termed the transmission efficiency, and can be defined at an individual level as the probability that an infectious bite leads to blood-stage infection, or at a population level as the proportion of bites on a population that cause new blood-stage infections. Smith *et al*. [16] reviewed studies reporting both the entomological inoculation rate (EIR) and the force of infection in children and noted that transmission efficiency decreased with increasing EIR. Several explanations for this effect have been put forward: heterogeneity in the force of infection [16]; an infection-blocking PE immune response [17,18]; density-dependent inhibition of sporozoite inoculation [19,20]; and inhibition of intrahepatic development by blood-stage parasites [21]. In addition, a strong blood-stage immune response that induces rapid clearance of parasites before detection can be conflated with a reduction in transmission efficiency [22].

Transmission efficiency is also dependent on age. In some, but not all, treatment re-infection studies, the time until detection of infection for adults has been observed to be significantly longer than for children, suggesting a reduction in transmission efficiency with age [15,23]. This effect has been observed despite the fact that mosquitoes bite adults more often than smaller children because of their larger surface area [24,25]. Smith [16] explained the variation in transmission efficiency in young children in terms of heterogeneity in the force of infection. If biting is homogeneous then infections are evenly distributed across the population resulting in a high transmission efficiency. If instead biting is heterogeneous, then transmission efficiency will be lower as most bites will be concentrated on highly exposed individuals who are likely to already be infected [26].

Heterogeneity in exposure, density-dependent inhibition of sporozoite inoculation and inhibition of intrahepatic development by blood-stage parasites cannot explain the difference in transmission efficiency between children and adults. The most common explanation for this reduction in transmission efficiency is the acquisition of PE immunity with age [17]. However, attempts to quantify the magnitude of the PE immune response have been complicated by apparently contradictory results from studies investigating the association between markers of immunity and protection from infection [27]. For example, the relationship between naturally acquired anti-CSP antibodies and protection from infection has been found to be positive [28], negative [29] and non-significant [30]. In order to explain these observations, a review of published studies of the relationship between PE immune responses and protection from infection or clinical malaria was conducted. The results of these studies are described in terms of their study design and sample size. In studies of PE immunity there is substantial variation in levels of the measured immune marker in the population cohort and hence no fixed effect size for protection from infection. Therefore standard methods using sample size calculations to estimate statistical power cannot be applied, necessitating the use of mathematical models to estimate the statistical power of studies to measure PE immunity.

## Methods

### Literature review

**Figure 1:**
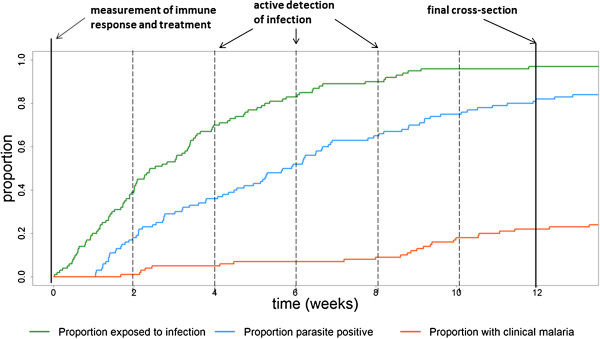
**Schematic representation of a treatment re-infection study for measuring pre-erythrocytic immune responses.** The solid black lines denote when samples are taken for a study with two cross-sections, and the dashed lines indicate when additional samples need to be taken for a longitudinal study. The difference between the cumulative proportion exposed to infection (green) and the cumulative proportion parasite positive (blue) is explained by pre-erythrocytic immunity and sporozoite inoculations that do not progress to blood-stage infection due to chance. The difference between the cumulative proportion parasite positive (blue) and the cumulative proportion with clinical malaria (red) is explained by blood-stage immunity.

A systematic search of the published literature was undertaken for studies investigating the association between markers of naturally acquired PE immunity and protection from malaria (Additional file 1: Table S1). The search was conducted using the online database PubMed with the terms “(malaria OR falciparum) AND (pre-erythrocytic OR infection-blocking)”. The results were supplemented by iterative reviews of the reference lists of relevant published papers. The primary aim was to investigate the association between PE immune responses and protection from *P. falciparum* infection. Each study was classified according to its design. In cross-sectional studies, participants were tested at the beginning and end of (and sometimes during) a study for parasitaemia and immune responses. In longitudinal studies, active detection of infection (ADI) was performed by testing participants for parasites at a given frequency. These study designs are summarized in Figure 1. Studies were further classified according to how immune response data were analysed, either as a binary variable (e g, high *vs* low responders, or sero-positive *vs* sero-negative), or a continuous variable (e g, antibody titre). Based on study design (cross-sectional/longitudinal) and classification of immune response (binary/continuous), the studies were classified into four categories.

1. **Cross-sectional study with binary immune response.** Participants are split into two categories according to measured immune responses, and infection is tested for at at least two cross-sections. A common study design involves clearing existing infections and taking a cross-section to test for new infections a number of weeks later. Tests for an association between the baseline immune response and protection from infection are then undertaken.

2. **Cross-sectional study with continuous immune response.** Similar to the previous study design except that immune responses are analysed as a continuous variable.

3. **Longitudinal study with ADI and binary immune response.** Participants are treated to clear existing infections and followed longitudinally with ADI at a given frequency. Immune responses, measured at the beginning of follow up, are used to classify participants into high and low categories. Survival analysis is used to test for an association between immune response and time to infection.

4. **Longitudinal study with ADI and continuous immune response.** Similar to the previous study design except that immune responses are analysed as a continuous variable.

There was much variation in the characteristics of the identified studies, with the most notable differences being in the endpoint under investigation, the study size and duration of follow up, the age range of the cohort and transmission intensity. With such variation in study characteristics it was not feasible to perform a meta-analysis of the effects of PE immune responses on protection from infection and therefore four study designs were simulated to estimate the dependence of statistical power on underlying covariates.

### Simulation of cross-sectional and longitudinal studies

**Figure 2:**
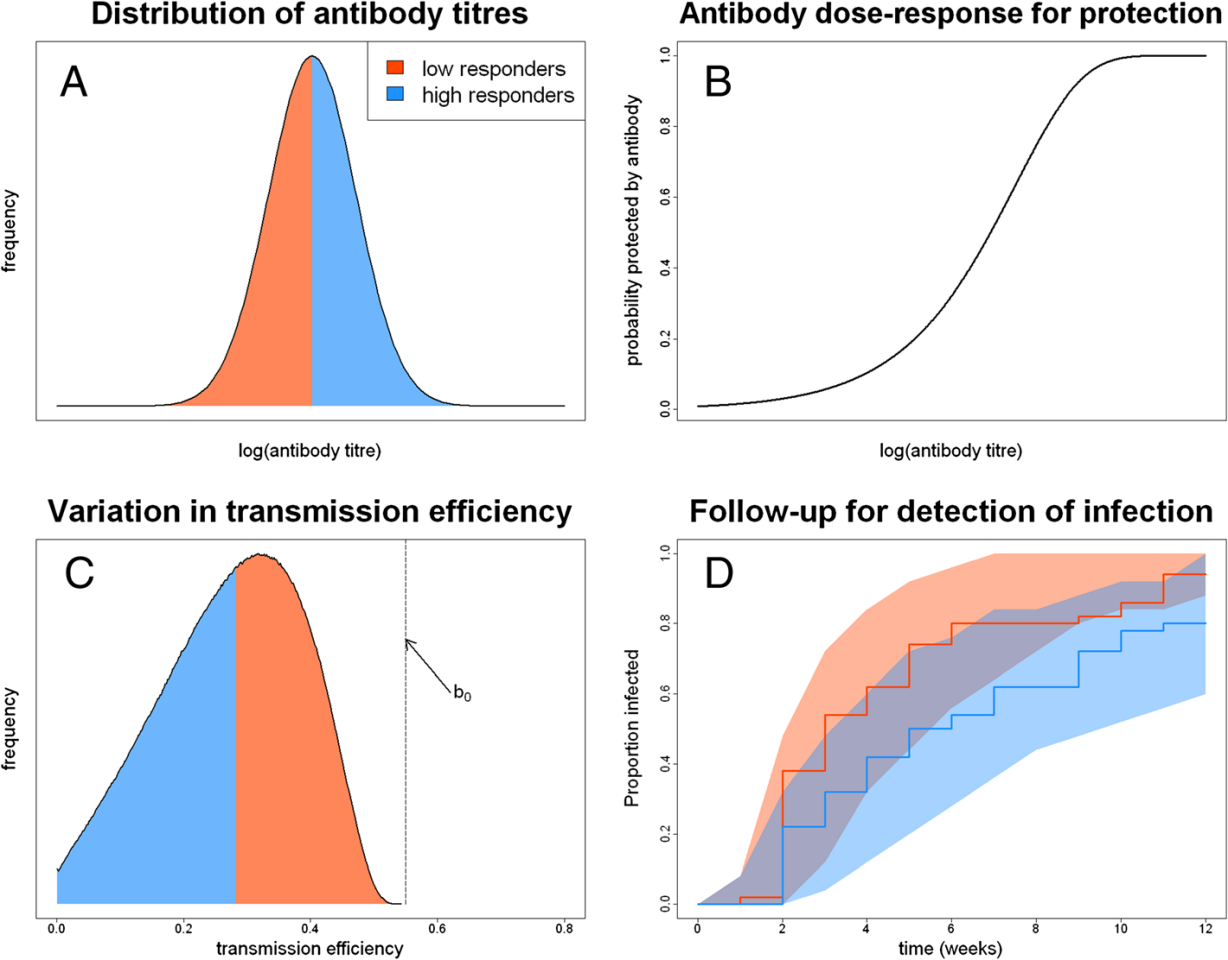
**Simulated longitudinal study for evaluation of the association between protection from infection and pre-erythrocytic antibodies.** It is assumed that 50 volunteers are followed for 12 weeks with active detection of infection. **(A)** Log-normal distribution of antibody titres in the study cohort, split into high (blue) and low (red) responders. **(B)** Dose–response curve used in model simulations for the probability of being protected by antibodies. **(C)** Distribution of transmission efficiency in the study population, split into high (blue) and low (red) responders. **(D)** Sample Kaplan-Meier curves for high and low responders. The red and blue shaded regions represent 95% confidence intervals for the KM curves of low and high responders, respectively. The substantial overlap between the shaded regions illustrates the low statistical power to differentiate between high and low responders.

Simulations were performed to demonstrate the effect of study size, duration of follow up, and transmission intensity on the statistical power to detect an effect of PE immunity. Transmission efficiency can be reduced by PE immune responses, as measured by some marker *α*. Markers of PE immunity can include antibody titre or measures of cell-mediated immunity such as numbers of antigen-specific T cells. In the absence of PE immunity, transmission efficiency was assumed equal to *b*_0_. It is assumed that PE immune marker *α* confers protection from infection according to an exponential dose–response curve $P\left(\alpha \right)=e^{-\log\left(2\right)\frac{\alpha }{\alpha_{50}}}$, where *α*_50_ confers 50% protection [31] (for example the antibody titre required to prevent 50% of infections). The transmission efficiency of a person with PE immune marker *α* is then reduced to *b*(*α*) = *b*_0_*P*(*α*). In a setting with EIR = *ε*, the force of infection is *Λ*(*α*) = *εb*_0_*P*(*α*). After clearance of any existing infection with anti-malarial drugs, the probability of being re-infected by time *t* is *I*(*t*) = 1 − *e*^− *Λ*(*α*)*t*^. The computer code for simulating this model was written in R and is included in Additional file 2. The outcome of a sample simulation, as well as the variation in antibody titres and force of infection in the study population is shown in Figure 2A-D. The parameters used for the simulations are given in Table 1.

**Table 1:** Parameters for simulation of treatment re-infection studies

**Description**	**Parameter**	**Value**
Baseline transmission efficiency	*b*_0_	0.55
PE marker needed to prevent 50% of infections	*α*_50_	1
Mean in population with 30% protection	*μ*_*α*_	0.56
Mean in population with 50% protection	*μ*_*α*_	1.19
Coefficient of variation in PE marker	*σ*_*α*_/*μ*_*α*_	0.75
Duration of follow-up (weeks)	T	12
Low transmission EIR (ibppy)	*ε*	5
High transmission EIR (ibppy)	*ε*	150

Baseline transmission efficiency based on estimates from Smith *et* al. [16]. Pre-erythrocytic immune markers are given in arbitrary dimensionless units and are assumed to be Log-Normally distributed across a population.

There are a number of limitations to this model. It is assumed that the force of infection is homogenous but the simulations can be adapted to incorporate heterogeneous biting. For simplicity, it is assumed that parasites are not cleared before detection, and submicroscopic infections and issues of sensitivity and specificity are ignored. These complexities are likely to further increase the uncertainty in identifying a significant relationship between immune markers and protection.

### Estimation of statistical power to detect the effect of pre-erythrocytic immunity

The effects of PE immunity in two hypothetical populations are considered, where a measured PE immune response causes an average 30 or 50% reduction in the force of infection compared to a population with no PE immunity (Table 1). Although the exact proportion of infections prevented by PE immune responses will be unknown prior to a study, these effect sizes covered a range of previously reported effect sizes [32]. Two trial designs are considered: a longitudinal study with weekly ADI for 12 weeks, and a cross-sectional study with cross-sections at the time of parasite clearance and 12 weeks later. The simulated data were analysed using Cox proportional hazards for time to infection in longitudinal studies with ADI, or logistic regression for the outcome (infected/not infected) in cross-sectional studies. Some 10,000 simulations were performed and the statistical power was estimated as the proportion of studies observing a statistical association between the PE immune marker and protection at the 5% significance level.

For the studies of the association between protection from infection and PE immunity identified in the systematic review, key design characteristics were recorded (Additional file 1: Table S1). The power of these studies to detect a statistically significant effect of a PE immune response preventing 30 or 50% of total infections in a population was estimated using simulations that matched the following study design characteristics: study size, duration of follow up, sampling frequency and estimated EIR.

## Results

### Review of studies of pre-erythrocytic immune responses

Nineteen studies of the association between markers of PE immunity and protection from infection were identified (Additional file 1: Table S1). Fifteen studies investigating the association between PE antibodies and protection from infection were identified; three of eleven found a statistically significant association between anti-CSP antibodies and protection at the 5% level. No studies found statistically significant associations between anti-TRAP or anti-LSA1 antibodies and protection. One study found a marginally significant association for anti-LSA1 antibodies [33], however this is likely due to correlation with other immune responses as LSA-1 epitopes are only expressed after hepatocyte invasion, at which stage immunoglobulin G (IgG) antibodies cease to be effective [34]. One of one studies found a statistically significant association between antibodies to multiple antigens and protection. Of the three studies detecting a significant association between PE antibodies and *P. falciparum* infection, two found antibodies to be associated with protection [32,35], and one found antibodies to be a risk factor for infection [29].

Eight studies investigating the association between cell-mediated immune (CMI) responses and protection were identified: One of two found a statistically significant association between CSP-specific CMI responses and protection, one of one found a statistically significant association between TRAP-specific CMI responses and protection, and two of four found a statistically significant association between LSA1-specific CMI responses and protection. One of one found a statistically significant association between combined CSP, TRAP and LSA1-specific CMI responses and protection. Of the eight studies detecting a significant association between markers of cell-mediated immunity and infection, five found these markers to be associated with protection, and one was found to be a risk factor for infection (Additional file 1: Table S1).

### Simulated effect of study design on statistical power

**Figure 3:**
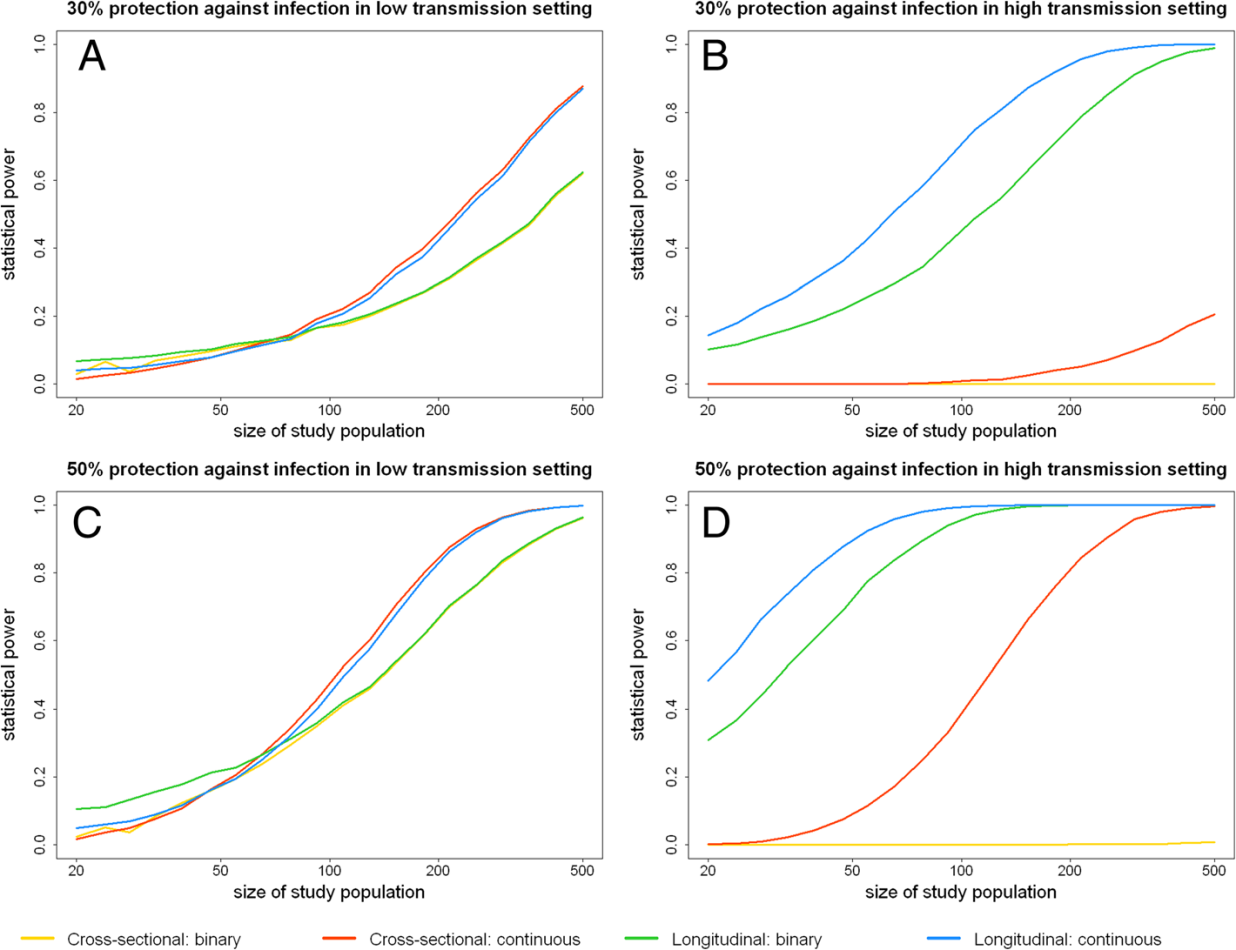
**Probability of observing a statistical association at the 5% significance level between a pre**-**erythrocytic immune response and protection from infection. (A)** A study in a low transmission setting (EIR = 5) where 30% of infections are prevented by pre-erythrocytic immunity. **(B)** A study in a high transmission setting (EIR = 150) where 30% of infections are prevented by pre-erythrocytic immunity. **(C)** A study in a low transmission setting (EIR = 5) where 50% of infections are prevented by pre-erythrocytic immunity. **(D)** A study in a high transmission setting (EIR = 150) where 50% of infections are prevented by pre-erythrocytic immunity.

The results of simulations to estimate the probability of detecting the effect of PE immunity at the 5% significance level are shown in Figure 3. Statistical power increases with study size, and there is a higher probability of detecting an effect if antibodies confer 50% protection from infection than if they confer 30% protection. Furthermore, greater statistical power is expected in high transmission settings (i e, with greater frequency of outcome) with studies implementing longitudinal follow up with ADI rather than a single cross-sectional survey at 12 weeks A cross-sectional design is expected to have lower statistical power in high transmission settings as it is likely that most participants will become infected between the baseline and final surveys.

Even with a longitudinal design utilizing a continuous measure of immunity, studies with 50 individuals have approximately 10% power to detect 30% protection against infection in low transmission settings (Figure 3A) and approximately 40% power in high transmission settings (Figure 3B). Even for a larger effect size where 50% of infections are prevented, a study of 50 individuals would be underpowered in a low transmission setting (Figure 3C). The simulation results suggest that cross-sectional surveys are underpowered to detect any likely association between immune responses and protection from infection except if the effect size is large (50% or more), the study is undertaken in a large population in a high transmission setting, and the immune response is considered as a continuous variable.

**Figure 4:**
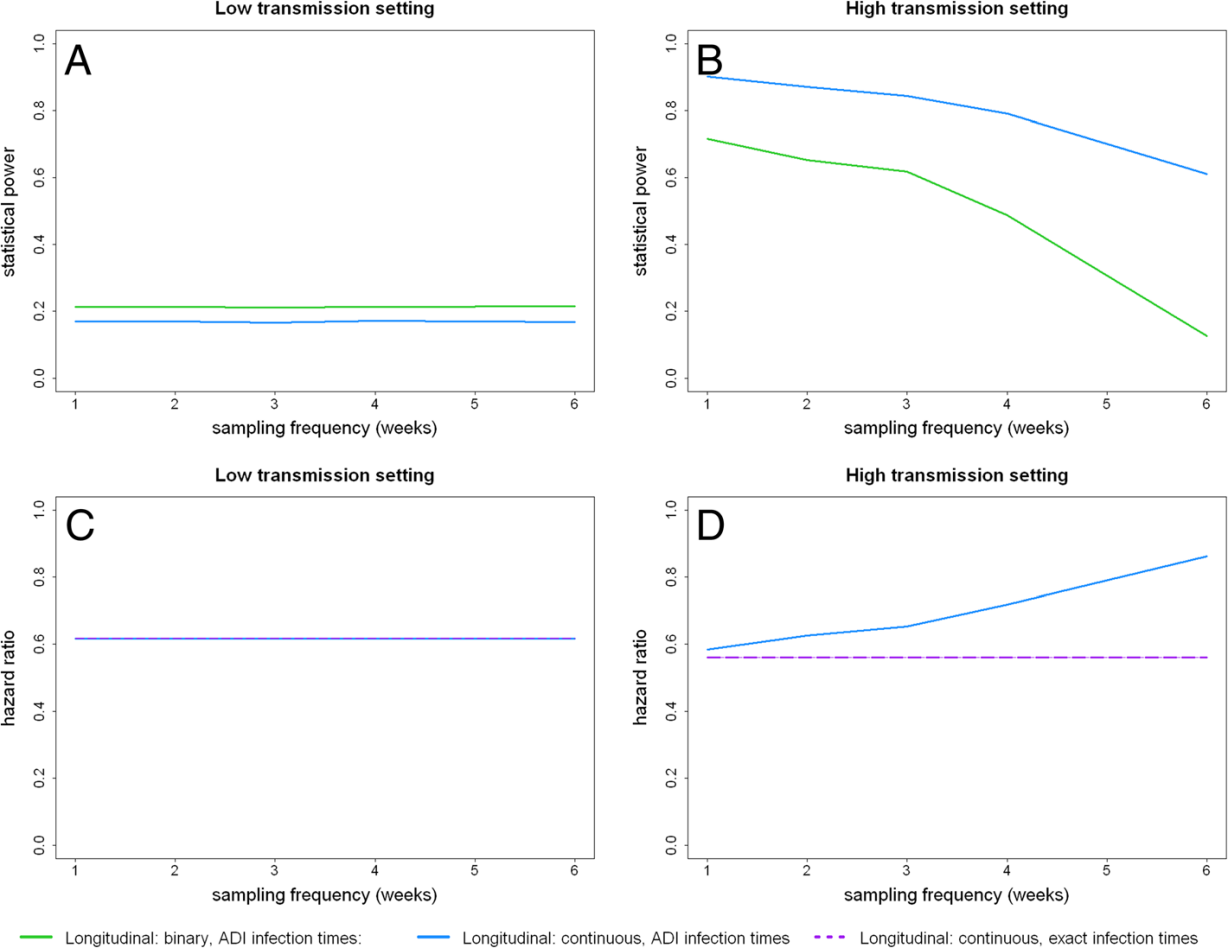
**The effect of sampling frequency (weeks between each evenly spaced sample assuming no seasonality in transmission) on statistical power and estimated hazard ratio.** Statistical power at the 5% significance level to detect the effect of a pre-erythrocytic immune response that prevents 50% of infections in **(A)** a low transmission setting (EIR = 5), and **(B)** a high transmission setting (EIR = 150). Mean of estimated hazard ratios for infection associated with a ten-fold increase in antibody titre in **(C)** a low transmission setting (EIR = 5), and **(D)** a high transmission setting (EIR = 150).

Figure 4 shows the statistical power of a longitudinal study with 50 participants as a function of sampling frequency. In a low transmission setting, the benefit of sampling more frequently than every six weeks is negligible as little additional information is obtained by testing volunteers who are unlikely to become infected (Figure 4A). In a high transmission setting, statistical power increases with increased sampling frequency (Figure 4B). As power increases slowly with sampling frequency, it may be most efficient to sample fortnightly or monthly if that would allow more volunteers to be recruited. In more intense transmission settings where all volunteers are likely to become infected, sampling frequency will need to be increased to accurately measure the time to re-infection.

In the studies reviewed, the time of infection is commonly assumed to be the time of detection by ADI, potentially introducing bias in the reduction in the hazard of infection with increased levels of PE immunity (Figures 4C and 4D). Increasing the sampling frequency will reduce this bias, although interval-censoring methods can be employed to correct for it. In low transmission settings where few participants are infected, this effect is limited (Figure 4C). However, in high transmission settings this can be appreciable if the sampling frequency is too low (Figure 4D). Selecting the optimal sampling frequency will depend on study size and duration, the expected magnitude of the immune response under investigation, transmission intensity and seasonality, the cost of processing samples and the need to avoid taking too many samples from any one person.

### Estimated statistical power of studies for measuring PE immune responses

**Figure 5:**
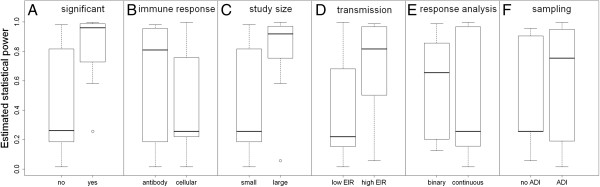
**Effect of different factors on the estimated statistical power to detect a pre-erythrocytic immune response that prevents 50% of infections for the study designs identified in the systematic review. (A)** Studies reporting statistically significant associations had higher estimated power than studies not reporting significant associations. **(B)** Studies of antibody responses had greater estimated power than studies of cellular responses. **(C)** Large studies (≥100 participants) had greater estimated power than small studies (< 100 participants). **(D)** Studies undertaken in high transmission settings (≥10 ibppy) had greater estimated power than studies undertaken in low transmission settings (< 10 ibppy). **(E)** Studies where the immune response was analysed as a binary variable had greater estimated statistical power than studies where responses were analysed as continuous variables. This result can be explained by the fact that studies with binary immune responses had larger numbers of participants than studies with continuous immune responses. **(F)** Studies with longitudinal follow up with active detection of infection (ADI) had greater estimated power than studies without ADI.

For the studies of the association between PE immune responses and protection from infection identified in the systematic review, model estimates of the statistical power at the 5% significance level to detect the effect of a PE immune response that prevents either 30 or 50% of infections were calculated (Additional file 1: Table S1). On average, studies reporting significant associations between PE immune responses and protection from infection had higher estimated power than studies not reporting significant associations (Figure 5A). Studies of antibody responses had higher estimated power than studies of CMI responses (Figure 5B), despite the fact that a higher proportion of studies of cellular immunity detected statistically significant associations than studies of antibodies (5/8 *vs* 3/15). The higher proportion of significant results in studies with lower estimated power suggests that cellular responses have a more important role in preventing infections than antibody responses [18].

Analysis of the estimated statistical power of the studies identified in the review demonstrates that larger studies were more likely to return significant results than smaller studies (Figure 5C), and that studies in high transmission settings were more likely to detect significant associations than studies in low transmission settings (Figure 5D). Studies where immune responses were analysed as binary variables had greater estimated power than studies where responses were analysed as continuous variables, although there was much variation (Figure 5E). This result can be explained by the fact that studies with binary immune responses had larger numbers of participants than studies with continuous immune responses. Finally, studies with longitudinal follow up with ADI had greater estimated power than cross-sectional studies without ADI (Figure 5F).

## Discussion

The PE immune response constitutes the first line of defence against the *P. falciparum* parasite, and will impact upon malaria transmission, the incidence of clinical malaria, and the efficacy of infection-blocking vaccines. Despites its key role in the epidemiology of malaria, the magnitude of the PE immune response remains poorly characterized and poorly quantified. In particular, identifying immune responses that significantly correlate with protection from infection has been difficult due to the low statistical power of treatment re-infection studies. Analysis of results from the literature demonstrates that the majority of studies failed to detect significant associations between PE immune responses and protection from infection. One interpretation of these findings is that PE immunity does not cause a meaningful reduction in *P. falciparum* infections. An alternative explanation is that PE immune responses prevent infections, but that studies are underpowered to consistently produce statistically significant findings. The results of model simulations demonstrates that increasing study size, undertaking studies in high transmission settings, implementing longitudinal follow-up with ADI, and analysing immunological data as a continuous variable will improve the probability of detecting an association between markers of PE immunity and protection from infection, if indeed such an association exists.

None of the studies identified in the review contained an estimate of statistical power, an important statistic for interpreting the results of epidemiological studies. Designing a study to investigate immune responses can be viewed as an optimization problem with the aim of maximizing the statistical power to detect an effect, and ensuring the effect size is estimated correctly, given real world limitations on time and resources. There will be trade-offs between the number of volunteers recruited, the sampling frequency, the duration of follow up, and the number of immune responses measured. The optimal study design will depend on numerous local characteristics such as seasonality and the age of participants, but in general, statistical power can be maximized by increasing the number of study participants, and performing the study in a high transmission setting. The least expensive thing to do to maximize statistical power is to fully utilize the immune response data by analysing it as a continuous rather than binary variable. Immunological data are often non-linearly associated with protection [36] so it may be beneficial to transform them before undertaking statistical analysis. Standard statistical methods such as logistic regression (for cross-sectional data) and proportional hazards methods (for longitudinal data) provide a convenient statistical framework for investigating the association between PE immunity and protection from infection, as it is straightforward to include interactions between multiple immune responses and to adjust for confounders such as LLIN use, age and location [37,38].

Longitudinal studies have advantages over cross-sectional studies in that they allow estimation of the incidence of infection and disease as opposed to just prevalence, and hence allow inferences of causality. Longitudinal studies also have greater statistical power than cross-sectional studies since they measure both who becomes infected and estimate the time of infection. Increasing the sampling frequency for ADI will increase statistical power but must be balanced by the cost of additional sampling. Most of the published longitudinal studies reviewed here performed survival analysis using the date of sampling when parasites were detected as the time of infection. However the true time of infection will have occurred at some time between the first positive sample and the previous negative sample. This approximation of the infection time results in biased estimates of the reduction in infections attributable to PE immune responses and can be corrected for by using interval-censored analysis to allow for the unknown infection time between consecutive samples.

One difficulty in investigating the association between individual immune responses and protection from malaria is that multiple immune responses can act cooperatively. A PE immune response to malaria is likely to be comprised of both antibody- and cell-mediated responses directed against multiple parasite antigens [39]. These responses are likely to be correlated as the acquisition of immunity depends on malaria exposure. Thus, if only a single immune response is measured, it may be acting as a marker for the entire immune response. Measuring multiple immune responses allows a more detailed investigation of the association between immunity and protection from malaria [32,40]. However, even if multiple responses are measured, protection may still be conferred via some other undetected immune response correlated with the measured response. A potential solution may be to use protein array analysis where plasma from individuals exposed to malaria is tested against a large proportion of the *P. falciparum* proteome [39,41], although this approach introduces additional difficulties due to multiple comparisons.

A second difficulty encountered in field studies is that immune responses can act as markers of exposure as well as correlates of protection [42] – as was seen when antibodies were found to be associated with protection in some studies and increased risk of infection in others. Heterogeneity in exposure will lead to heterogeneity in the rate of acquisition of immunity, with individuals under the most intense exposure developing the strongest immune response. Individuals identified as having a strong immune response may be at increased risk of future infection compared to the rest of the cohort as their increased exposure may outweigh the benefits of the stronger response. This phenomenon may lead to a study identifying protective antibodies as a risk factor for future infection [38].

Heterogeneity in exposure, the potential for immune responses to act as markers for exposure, and the possibility of correlation between multiple immune responses constitute limitations to the model used for the estimation of statistical power, and hence the results presented here. A constant force of infection was assumed, which equates to an exponential distribution for time to re-infection. The model may also be adapted to capture seasonality in transmission and hence better reflect real world conditions. Time-dependent covariates may be used to test for a changing effect over time. Furthermore, if multiple measures of PE immune response are available at different time points then they may be analysed as time-dependent covariates, as markers of naturally-acquired immunity measured at baseline may not correlate well with the risk of infection in the latter part of the study, as several reports indicate naturally acquired *P. falciparum* specific responses are relatively short-lived [43,44]. The assumption of a constant force of infection in proportional hazards models is violated by the heterogeneous distribution of infectious bites. Unaccounted heterogeneity in the force of infection can lead to biased estimates of the hazard rate [26], although this can be ameliorated by using survival analysis methods incorporating a frailty distribution to account for unmeasured heterogeneity.

## Conclusion

The results of studies to investigate the association between markers of PE immunity and protection from infection have often been inconclusive due to sub-optimal study design. The power to detect an effect of a PE immune response can be increased by increasing study size, undertaking studies in high transmission settings, performing longitudinal follow up with ADI and analysing immune responses as a continuous variable.

## Competing interests

The authors declare that they have no competing interests.

## Authors’ contributions

MTW and ACG devised the study. MTW and JTG developed the statistical methods. MTW prepared the manuscript. All authors read and approved the final manuscript.

## Supplementary Material

Additional file 1: Table S1 Review of quantitative studies of the relationship between pre-erythrocytic immune responses and protection from Plasmodium falciparum infection. Click here for file

Additional file 2 Code for estimating the statistical power of longitudinal or cross-sectional trials for measuring the effect of markers of pre-erythrocytic immunity. Click here for file
