# Supplementary material for: The design and statistical power of treatment re-infection studies of the association between pre-erythrocytic immunity and infection with Plasmodium falciparum
Source: Malar J. 2013 Aug 8;12:278. doi: 10.1186/1475-2875-12-278 (PMC3751675; doi:10.1186/1475-2875-12-278)
Supplement: Additional file 1: Table S1 — Review of quantitative studies of the relationship between pre-erythrocytic immune responses and protection from Plasmodium falciparum infection. [file 1475-2875-12-278-S1.docx]

Supplementary Table 1: Review of quantitative studies of the relationship between pre-erythrocytic immune responses and protection from *Plasmodium falciparum* infection. NS = not significant. ADI = active detection of infection. HR = hazard ratio. RR = relative risk. The last two columns show our estimate of the power to detect an effect, under the assumptions described in the main text†.

| **Reference** | **Cohort** | **Immune marker** | **Study design** | **Response** | **Statistical test** | **Transmission** | **Effect size** | **Significance** | **Power: 30%** | **Power: 50%** |
| --- | --- | --- | --- | --- | --- | --- | --- | --- | --- | --- |
| John 2005 [[1](#_ENREF_1)] | Nyanza, Kenya  77 adults | CSP IgG | weekly ADI for 12 weeks | continuous | Cox PH | holoendemic | HR=0.65 | 0.009 (68) | 0.53 | 0.97 |
|  |  | TRAP IgG |  | continuous | Cox PH |  | HR=0.70 | 0.17 (68) | 0.53 | 0.97 |
|  |  | LSA-1 IgG |  | binary | Cox PH |  | HR=0.72 | NS (68) | 0.53 | 0.97 |
|  |  | CSP TRAP LSA-1 IgG |  | binary | Cox PH |  | HR=0.43 | 0.006 (68) | 0.53 | 0.97 |
|  |  |  |  |  |  |  |  |  |  |  |
| Perraut 2003 [[2](#_ENREF_2)] | Ndiop, Senegal  110 all age groups | CSP IgG | weekly ADI for 12 weeks | binary | Cox PH | mesoendemic | **–** | 0.976 (110) | 0.41 | 0.86 |
|  |  |  |  |  |  |  |  |  |  |  |
| Flanagan 2003 [[3](#_ENREF_3)] | Kilifi, Kenya  217, all ages | TRAP IFNg | weekly ADi for 6 months | binary | Cox PH | mesoendemic | RR=2.30 | 0.05 (138) | 0.26 | 0.58 |
|  |  |  |  |  |  |  |  |  |  |  |
| John 2003 [[4](#_ENREF_4)] | Uasin Gishu, Kenya 71 adults | CSP IgG | weekly ADI for 10 weeks | binary | Cox PH | hypoendemic | HR=0.92 | 0.887 (50) | 0.10 | 0.19 |
|  |  | TRAP IgG |  | binary | Cox PH |  | HR=1.41 | 0.565 (50) | 0.10 | 0.19 |
|  |  | LSA-1 IgG |  | binary | Cox PH |  | HR=2.11 | 0.157 (50) | 0.10 | 0.19 |
|  | Uasin Gishu, Kenya, 37 children | CSP IgG |  | binary | Cox PH |  | HR=2.06 | 0.295 (32) | 0.08 | 0.13 |
|  |  | TRAP IgG |  | binary | Cox PH |  | HR=0.13 | 0.108 (32) | 0.08 | 0.13 |
|  |  |  |  |  |  |  |  |  |  |  |
| Ong’echa 2003 [[5](#_ENREF_5)] | Asembo Bay, Kenya 107 children | CSP TRAP LSA-1 proliferative response | ADI | binary | Chi-square | holoendemic | **–** | 0.007 (107) | 0.01 | 0.73 |
|  |  | CSP TRAP LSA-1 IFNg |  | binary | Chi-square | holoendemic | **–** | 0.252 (107) | 0.01 | 0.73 |
|  |  |  |  |  |  |  |  |  |  |  |
| John 2002 [[6](#_ENREF_6)] | Uasin Gishu, Kenya children and adults | LSA-1 IgG | weekly ADI for 10 weeks | binary | Cox PH | hypoendemic | **–** | NS (93) | 0.14 | 0.31 |
|  |  | LSA-1 cell proliferation |  | binary | Cox PH |  | **–** | NS (57) | 0.11 | 0.20 |
|  |  |  |  |  |  |  |  |  |  |  |
| John 2000 [[7](#_ENREF_7)] | Uasin Gishu, Kenya  Children and adults | LSA-1 IL5 | weekly ADI for 10 weeks | binary | Cox PH | hypoendemic | RR=0.84 | NS (69) | 0.12 | 0.24 |
|  |  | LSA-1 IL10 |  | binary | Cox PH |  | RR=0.51 | 0.083 (77) | 0.13 | 0.26 |
|  |  | LSA-1 IFNg |  | binary | Cox PH |  | RR=0.99 | NS (94) | 0.14 | 0.31 |
|  |  | LSA-1 TNFa |  | binary | Cox PH |  | RR=0.86 | NS (54) | 0.10 | 0.19 |
|  |  |  |  |  |  |  |  |  |  |  |
| Domarle 1999 [[8](#_ENREF_8)] | Dienga, Gabon 61 children | LSA-Rep IgG | weekly ADI for 11 weeks, then every 2 weeks up to week 30 | binary | Mann-Whitney | hyperendemic | **–** | 0.08 (61) | 0.30 | 0.82 |
|  |  | LSA-J IgG |  | binary | Mann-Whitney |  | **–** | 0.051 (61) | 0.30 | 0.82 |
|  |  | CSP IgG (NANP) |  | binary | Mann-Whitney |  | **–** | 0.82 (61) | 0.30 | 0.82 |
|  |  | CSP IgG (NAAG) |  | binary | Mann-Whitney |  | **–** | 0.84 (61) | 0.30 | 0.82 |

Supplementary Table 1 (contd.): Review of quantitative studies of the relationship between pre-erythrocytic immune responses and protection from *Plasmodium falciparum* infection. NS = not significant. ADI = active detection of infection. HR = hazard ratio. RR = relative risk. The last two columns show our estimate of the power to detect an effect, under the assumptions described in the main text†.

| **Reference** | **Cohort** | **Immune marker** | **Study design** | **Response** | **Statistical test** | **Transmission** | **Effect size** | **Significance** | **Power: 30%** | **Power: 50%** |
| --- | --- | --- | --- | --- | --- | --- | --- | --- | --- | --- |
| Kurtis 1999 [[9](#_ENREF_9)] | Nyanza, Kenya  178 adults | LSA-1 IL10 | weekly ADI for 16 weeks | binary | Cox PH | holoendemic | RR=0.63 | 0.05 (137) | 0.41 | 0.92 |
|  |  |  |  | continuous | Cox PH |  | **–** | 0.03 (137) | 0.49 | 0.99 |
|  |  |  |  |  |  |  |  |  |  |  |
| Connelly 1997 [[10](#_ENREF_10)] | Papua New Guinea  55 adults | LSA-1 IFNg (84-107) | cross-section at 6 month intervals | continuous | Student t-test | holoendemic | RR=0.27 | <0.001 (38) | 0.01 | 0.26 |
|  |  | LSA-1 IFNg (1813-1835) |  | continuous | Student t-test |  | RR=0.91 | NS (38) | 0.01 | 0.26 |
|  |  | LSA-1 IFNg (1888-1909) |  | continuous | Student t-test |  | RR=1.00 | NS (38) | 0.01 | 0.26 |
|  |  | LSA-1 IgG (84-107) |  | continuous | Student t-test |  | RR=1.43 | NS (38) | 0.01 | 0.26 |
|  |  | LSA-1 IgG (1813-1835) |  | continuous | Student t-test |  | RR=1.75 | NS (38) | 0.01 | 0.26 |
|  |  | LSA-1 IgG (1888-1909) |  | continuous | Student t-test |  | RR=1.40 | NS (38) | 0.01 | 0.26 |
|  |  |  |  |  |  |  |  |  |  |  |
| Scarselli 1993 [[11](#_ENREF_11)] | Mali  66 children | TRAP IgG | bi-weekly ADI for 30 weeks | continuous | PH linear regression | hyperendemic | RR=0.15 | NS (66) | 0.24 | 0.87 |
|  |  |  |  |  |  |  |  |  |  |  |
| Deloron 1991 [[12](#_ENREF_12)] | Madagascar  40 adults | CSP IgG | weekly ADI | continuous | Mann-Whitney | hyperendemic | RR=0.68 | 0.20 (33) | 0.02 | 0.02 |
|  |  | CSP cell proliferation |  | continuous | Mann-Whitney |  | RR=1.11 | 0.38 (28) | 0.02 | 0.02 |
|  |  | CSP IFNg |  | continuous | Mann-Whitney |  | RR=0.15 | 0.05 (33) | 0.02 | 0.02 |
|  |  |  |  |  |  |  |  |  |  |  |
| Wongsrichanalai 1991 [[13](#_ENREF_13)] | Thai adults | CSP IgG | case-control, monthly ADI | binary |  | hypoendemic | RR=1.77 | NS (132) | 0.36 | 0.78 |
|  |  |  |  |  |  |  |  |  |  |  |
| Rooth 1991 [[14](#_ENREF_14)] | Rufiji, Tanzania 68 children | CSP IgG | ADI weekly for 4 weeks | Binary | Student t-test | holoendemic | **–** | NS (68) | 0.24 | 0.80 |
|  |  |  |  |  |  |  |  |  |  |  |
| Riley 1990 [[15](#_ENREF_15)] | Farafenni, Gambia, 391 children | CSP IFNg (Th2R) | weekly ADI | continuous | logistic regression | mesoendemic | RR=0.46 | NS | 0.62 | 0.97 |
|  |  | CSP IFNg (Th3R) |  | continuous | logistic regression |  | RR=0.28 | 0.04 | 0.62 | 0.97 |
|  |  |  |  |  |  |  |  |  |  |  |
| Snow 1989 [[16](#_ENREF_16)] | Farafenni, Gambia, 376 children | CSP IgG | 2 cross sections | binary | Mantel-Haensel | mesoendemic | RR=1.32 | P<0.01 (292) | 0.59 | 0.96 |
|  |  |  |  | continuous | ANOVA |  | **–** | NS (292) | 0.59 | 0.96 |
|  |  |  |  |  |  |  |  |  |  |  |
| Chizzolini 1988 [[17](#_ENREF_17)] | Gabon, all ages | CSP IgG | 2 cross sections | continuous | Student t-test | mesoendemic | RR=1.18 | NS (144) | 0.01 | 0.06 |

Supplementary Table 1 (contd.): Review of quantitative studies of the relationship between pre-erythrocytic immune responses and protection from *Plasmodium falciparum* infection. NS = not significant. ADI = active detection of infection. HR = hazard ratio. RR = relative risk. The last two columns show our estimate of the power to detect an effect, under the assumptions described in the main text†.

| **Reference** | **Cohort** | **Immune marker** | **Study design** | **Response** | **Statistical test** | **Transmission** | **Effect size** | **Significance** | **Power: 30%** | **Power: 50%** |
| --- | --- | --- | --- | --- | --- | --- | --- | --- | --- | --- |
| Marsh 1988 [[18](#_ENREF_18)] | Farafenni, Gambia, children 1-11 years | CSP IgG | 2 cross sections, weekly morbidity survey | binary | Chi-square | mesoendemic | RR=0.34 | 0.01 (124) | 0.16 | 0.91 |
|  | Farafenni, Gambia, adults 12+ | CSP IgG |  | binary | Chi-square |  | RR=0.56 | NS (64) | 0.02 | 0.50 |
|  |  |  |  |  |  |  |  |  |  |  |
| Hoffman 1987 [[19](#_ENREF_19)] | Saradidi, Kenya  83 adults | CSP IgG | ADI every 2 weeks for 14 weeks | continuous | Linear regression | holoendemic | **–** | NS (83) | 0.55 | 0.98 |

**†**The estimated statistical power is dependent on the model assumptions outlined in the text and the assumed magnitude of the effect size to be estimated. The actual statistical power of the studies reviewed here will depend on characteristics such as heterogeneity in exposure and correlation with other immune responses not captured in our simple model.

**References**

1. John CC, Moormann AM, Pregibon DC, Sumba PO, McHugh MM, Narum DL, Lanar DE, Schluchter MD, Kazura JW: **Correlation of high levels of antibodies to multiple pre-erythrocytic Plasmodium falciparum antigens and protection from infection**. *American Journal of Tropical Medicine and Hygiene* 2005, **73**(1):222-228.

2. Perraut R, Marrama L, Diouf B, Fontenille D, Tall A, Sokhna C, Trape JF, Garraud O, Mercereau-Puijalon O: **Distinct surrogate markers for protection against Plasmodium falciparum infection and clinical malaria identified in a Senegalese community after radical drug cure**. *Journal of Infectious Diseases* 2003, **188**(12):1940-1950.

3. Flanagan KL, Mwangi T, Plebanski M, Odhiambo K, Ross A, Sheu E, Kortok M, Lowe B, Marsh K, Hill AVS: **Ex vivo interferon-gamma immune response to thrombospondin-related adhesive protein in coastal Kenyans: Longevity and risk of Plasmodium falciparum infection**. *American Journal of Tropical Medicine and Hygiene* 2003, **68**(4):421-430.

4. John CC, Zickafoose JS, Sumba PO, King CL, Kazura JW: **Antibodies to the Plasmodium falciparum antigens circumsporozoite protein, thrombospondin-related adhesive protein, and liver-stage antigen 1 vary by ages of subjects and by season in a highland area of Kenya**. *Infection and Immunity* 2003, **71**(8):4320-4325.

5. Ong'echa JMO, Lal AA, Terlouw DJ, Ter Kuile FO, Kariuki SK, Udhayakumar V, Orago ASS, Hightower AW, Nahlen BL, Shi YP: **Association of interferon-gamma responses to pre-erythrocytic stage vaccine candidate antigens of Plasmodium falciparum in young Kenyan children with improved hemoglobin levels: XV. Asembo Bay Cohort Project**. *American Journal of Tropical Medicine and Hygiene* 2003, **68**(5):590-597.

6. John CC, Ouma JH, Sumba PO, Hollingdale MR, Kazura JW, King CL: **Lymphocyte proliferation and antibody responses to Plasmodium falciparum liver-stage antigen-1 in a highland area of Kenya with seasonal variation in malaria transmission**. *American Journal of Tropical Medicine and Hygiene* 2002, **66**(4):372-378.

7. John CC, Sumba PO, Ouma JH, Nahlen BL, King CL, Kazura JW: **Cytokine responses to Plasmodium falciparum liver-stage antigen 1 vary in rainy and dry seasons in highland Kenya**. *Infection and Immunity* 2000, **68**(9):5198-5204.

8. Domarle O, Migot-Nabias F, Mvoukani JL, Lu CY, Nabias R, Mayombo J, Tiga H, Deloron P: **Factors influencing resistance to reinfection with Plasmodium falciparum**. *American Journal of Tropical Medicine and Hygiene* 1999, **61**(6):926-931.

9. Kurtis JD, Lanar DE, Opollo M, Duffy PE: **Interleukin-10 responses to liver-stage antigen 1 predict human resistance to Plasmodium falciparum**. *Infection and Immunity* 1999, **67**(7):3424-3429.

10. Connelly M, King CL, Bucci K, Walters S, Genton B, Alpers MP, Hollingdale M, Kazura JW: **T-cell immunity to peptide epitopes of liver-stage antigen 1 in an area of Papua New Guinea in which malaria is holoendemic**. *Infection and Immunity* 1997, **65**(12):5082-5087.

11. Scarselli E, Tolle R, Koita O, Diallo M, Muller HM, Fruh K, Doumbo O, Crisanti A, Bujard H: **Analysis of the human-antibody response to thrombospondin-related anonymous protein of Plasmodium-falciparum**. *Infection and Immunity* 1993, **61**(8):3490-3495.

12. Deloron P, Chougnet C, Lepers JP, Tallet S, Coulanges P: **Protective value of elevated levels of gamma-interferon in serum against exoerythrocytic stages of Plasmodium-falciparum**. *Journal of Clinical Microbiology* 1991, **29**(9):1757-1760.

13. Wongsrichanalai C, Webster HK, Permpanich B, Chuanak N, Ketrangsri S: **Naturally acquired circumsporozoite antibodies and their role in protection in endemic falciparum and vivax malaria**. *American Journal of Tropical Medicine and Hygiene* 1991, **44**(2):201-204.

14. Rooth I, Perlmann H, Bjorkman A: **Plasmodium-falciparum reinfection in children from a holoendemic area in relation to seroreactivities against oligopeptides from different malaria antigens**. *American Journal of Tropical Medicine and Hygiene* 1991, **45**(3):309-318.

15. Riley EM, Allen SJ, Bennett S, Thomas PJ, Odonnell A, Lindsay SW, Good MF, Greenwood BM: **Recognition of dominant T cell-stimulating epitopes from the circumsporozoite protein of Plasmodium-falciparum and relationship to malaria morbidity in Gambian children**. *Transactions of the Royal Society of Tropical Medicine and Hygiene* 1990, **84**(5):648-657.

16. Snow RW, Shenton FC, Lindsay SW, Greenwood BM, Bennett S, Wheeler J, Delgiudice G, Verdini AS, Pessi A: **Sporozoite antibodies and malaria in children in a rural area of the Gambia**. *Annals of Tropical Medicine and Parasitology* 1989, **83**(6):559-568.

17. Chizzolini C, Dupont A, Akue JP, Kaufmann MH, Verdini AS, Pessi A, Delgiudice G: **Natural antibodies against 3 distinct and defined antigens of Plasmodium-falciparum in residents of a mesoendemic area in Gabon**. *American Journal of Tropical Medicine and Hygiene* 1988, **39**(2):150-156.

18. Marsh K, Hayes RH, Carson DC, Otoo L, Shenton F, Byass P, Zavala F, Greenwood BM: **Anti-sporozoite antibodies and immunity to malaria in a rural Gambian population**. *Transactions of the Royal Society of Tropical Medicine and Hygiene* 1988, **82**(4):532-537.

19. Hoffman SL, Oster CN, Plowe CV, Woollett GR, Beier JC, Chulay JD, Wirtz RA, Hollingdale MR, Mugambi M: **Naturally acquired antibodies to sporozoites do not prevent malaria - vaccine development implications**. *Science* 1987, **237**(4815):639-642.
